# Age-variant and age-invariant features of functional brain organization in middle-aged and older autistic adults

**DOI:** 10.1186/s13229-020-0316-y

**Published:** 2020-01-22

**Authors:** Joe Bathelt, P. Cédric Koolschijn, Hilde M. Geurts

**Affiliations:** Dutch Autism & ADHD Research Center, Brain & Cognition, Department of Psychology, University of Amsterdam, Nieuwe Achtergracht 129-B, 1018 WS Amsterdam, Netherlands

**Keywords:** Aging, Autism spectrum disorder, Graph theory, Functional connectivity

## Abstract

**Background:**

The majority of research effort into autism has been dedicated to understanding mechanisms during early development. As a consequence, research on the broader life course of an autism spectrum condition (ASC) has largely been neglected and almost nothing is known about ASC beyond middle age. Differences in brain connectivity that arise during early development may be maintained across the lifespan and may play protective or detrimental roles in older age.

**Method:**

This study explored age-related differences in functional connectivity across middle and older age in clinically diagnosed autistic adults (*n* = 44, 30–73 years) and in an age-matched typical comparison group (*n* = 45).

**Results:**

The results indicated parallel age-related associations in ASC and typical aging for the local efficiency and connection strength of the default mode network and for the segregation of the frontoparietal control network. In contrast, group differences in visual network connectivity are compatible with a safeguarding interpretation of less age-related decline in brain function in ASC. This divergence was mirrored in different associations between visual network connectivity and reaction time variability in the ASC and comparison group.

**Limitations:**

The study is cross-sectional and may be affected by cohort effects. As all participants received their autism diagnosis in adulthood, this might hinder generalizability.

**Conclusion:**

These results highlight the complexity of aging in ASC with both parallel and divergent trajectories across different aspects of functional network organization.

## Introduction

An autism spectrum condition (ASC)^1^ is a neurodevelopmental condition that is commonly characterized by impairments in social interaction, social communication, and restricted and stereotyped behaviors and interests (American Psychiatric Association 2013). The earliest signs of ASC emerge early in life, typically in infancy. While the nature of symptoms may change with increasing chronological and developmental age, ASC is considered a chronic condition with no known spontaneous remissions. Hitherto, most research has focused on children and adolescents with an ASC, but much less is known about the lifespan trajectory of ASC beyond early to mid-adulthood. It is vital to develop a more complete understanding of aging in individuals with ASC to better address their needs in old age and to distinguish typical aging in autistic adults from age-related disorders like dementia.

There are some indications that aging is of particular concern for people with ASC. First, the epidemiological evidence suggests a two- to three-fold increase in the mortality rate of middle-aged adults with ASC [2, 3]. Second, older adults with an ASC report more cognitive failures in everyday life [4]. Yet, cognitive assessments show little evidence of a steeper age-related decline in ASC [4]. A potential reason for this discrepancy is the limitation of lab-based assessments of cognitive function [5].While older individuals may have learned to compensate for difficulties on laboratory tasks, their neurocognitive systems may struggle with the complex demands of everyday situations. Non-invasive neuroimaging methods like functional MRI (fMRI) provide an insight into brain mechanisms that are difficult to distinguish at a behavioral level and so-called resting-state fMRI (rsfMRI) reproduces the same large-scale functional networks that are also picked up by fMRI under cognitive tasks [6]. A large body of literature documents differences in rsfMRI connectivity in children and adolescents with ASC [7]. The current synthesis of this literature suggests a pattern of altered segregation and integration characterized by local hyper-connectivity and global hypoconnectivity in ASC [8, 9]. Furthermore, reduced connectivity of nodes within the default mode network (DMN) and between the DMN and other functional networks is a consistent finding [10–12]. These brain-level differences have been found to be associated with cognitive differences in social processing and executive function [13, 14]. It is not currently known if these differences in functional brain organization and their association with cognitive differences are maintained across the lifespan and what role they may play in older age.

Several candidate accounts of age-related trajectories in ASC have been put forward [15]. On the one hand, neural and cognitive differences in ASC may be maintained across the lifespan and follow the same age-related decline as in typical individuals (*parallel development hypothesis*). On the other hand, individuals with an ASC may be predisposed to a more rapid age-related decline (*accelerated aging hypothesis*), either due to mechanisms that are specific to aging in ASC or due to increased vulnerability associated with ASC that lead to accelerated aging, e.g., because of differences in lifestyle. Similar accelerated aging has been suggested in other neurocognitive disorders, most notable schizophrenia [16]. Biological processes or differences in cognition or lifestyle associated with ASC may also protect against age-related decline (*safeguard hypothesis*), e.g., at the biological level because of protective effects afforded by cortical hyperplasticity in ASC [17] or redundancies in network connections [18] as theoretical work and investigations in other syndromes suggest. The current study set out to explore age-related differences in rsfMRI in individuals with an ASC and a typical comparison group across the whole brain and within functional networks. To characterize functional network organization, we focused on graph theoretical measures and comparisons of large-scale functional networks that have been implicated in aging and ASC research. We explored whether we observe indications for either parallel age-related trajectories or an increased or decreased age-related decline in ASC. Further, we expected a relationship between functional brain organization and cognitive assessments that are sensitive to ASC and aging, specifically reaction time variability and social processing.

## Methods and materials

### Participants

The study was carried out in agreement with the Declaration of Helsinki. All participants provided written informed consent. The study was approved by the university ethics reviewer board (#2013-PN-2668). Fifty-one individuals with an ASC (Age [means ± SD]: 45.9 ± 13.71 years, 35 male) and 49 comparison individuals without ASC (CMP group; age [means ± SD]: 50.1 ± 11.81 years, 32 male) between 30 and 74 years were recruited from a cohort of participants (estimated IQ > 80) of a large-scale behavioral study [4, 19, 20]. Details on inclusion criteria have been described earlier [20]. In short, all autistic individuals received their clinical ASC diagnosis by a multidisciplinary specialist team. To further ascertain the ASC diagnosis, the following inclusion criteria were applied: (1) formal clinical diagnosis of ASC prior to inclusion; (2) confirmation of diagnosis with the Autism Diagnostic Observation Schedule Module 4 [21] and/or Autism-Spectrum Quotient (50-item list, [22]). According to the clinical cut-offs, 31 individuals scored above the critical Autism Diagnostic Observation Schedule (ADOS) score (≥ 7) and those who did not score above this threshold did score above the clinical cut-off on the autism spectrum quotient AQ (≥ 26) (also see [23, 24] for similar approaches). (3) No self-reported history of neurological disorders, chronic illness, learning disabilities, or schizophrenia. Participants in the comparison group also had to meet this criterion. (4) Participants in the comparison group could not have an ASC diagnosis or a first or second-degree family member with ASC. Seven participants in the ASC group and four in the CMP group were excluded due to low-quality fMRI data (see Additional file 1 for quality control) leaving a final sample of 44 ASC and 45 CMP. We did not find evidence for between-group differences in full-scale IQ or age, nor differences in the sex or handedness ratio per group (see Table 1).

**Table 1:**
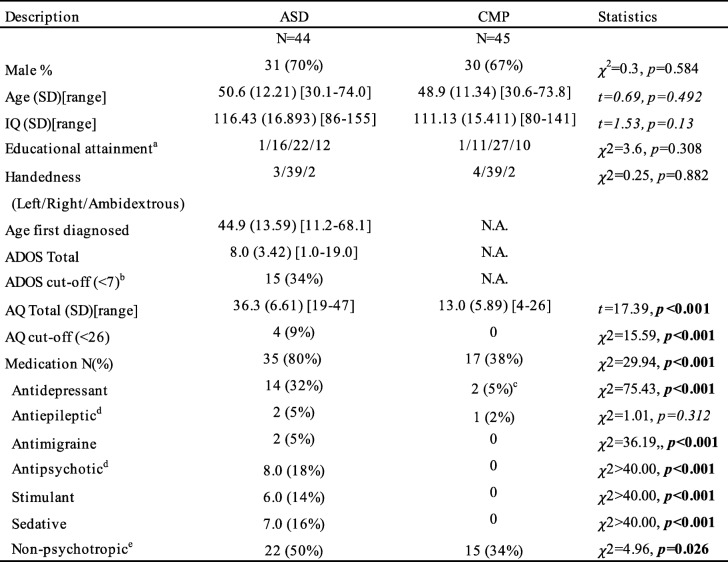
Characteristics of the ASC and CMP groups. Note: Numbers in bold reflect significant between group differences

### Data acquisition and pre-processing

MR data were acquired on a 3-T Achieva TX scanner (Philips Medical Systems, Best, The Netherlands) using a 32-channel head coil. Functional imaging data were acquired in a resting-state and two task paradigms all using a gradient-echo, echo-planar pulse sequence covering the whole brain (TR = 2000 ms; TE = 2763 ms; FA = 76.1°; 37 axial slices with ascending acquisition; 3 mm × 3 mm × 3.3 mm voxel size; 80 × 80 matrix; 240 × 121.80 × 240 FoV). A high-resolution 3D T1-weighted image was also acquired for spatial normalization (TR = 8.506 ms; TE = 3.94 ms; FA = 8°; 1 mm^3^ voxel size; 240 × 220 × 188 FoV). Participants were trained in a mock-scanner, were instructed to lie still during the scan, and to not fall asleep. None of the participants fell asleep during the scan. Head motion was further restricted with foam inserts around the head. Pre-processing of the T1-weighted and fMRI sequences was carried out using *fmriprep* v1.2.1 [25]. The details of the pre-processing pipelines are available in the Additional file 1. The code for all parts of the analysis is available online (Link: Open Science Framework).

### Functional connectivity analysis

The functional connectome was calculated as the Pearson correlation between time series within regions of interest (ROI). ROIs were defined according to a meta-analytic parcellation that identified independent functional regions [26]. ROIs that did not contain sufficient signal due susceptibility artifacts were removed (total remaining: 237, see Additional file 1). A minimum fMRI acquisition length of more than 20 min is required to estimate stable individual features of functional connectivity [27–29]. The current analysis relied on pre-collected data from a short resting-state acquisition (~ 5 min). To obtain sufficient data, functional connectivity from a resting-state sequence was combined with general functional connectivity from two task sequences [30, 31]. One task was a social processing paradigm in which participants had to discriminate faces from face-like Mooney images [32]. The other task was an Eriksen Flanker-type response inhibition paradigm [33], see Additional file 1 for detailed task descriptions). Both tasks were selected as autistic individuals are thought to perform differently on tasks related to (a) perceptual processing such as face processing and (b) executive functions such as inhibitory control [34]. To obtain generalized functional connectivity from the task fMRI data, the task-related activity was regressed from the task fMRI time series data as described in [30]. Using this procedure, more than 20 min of good quality data could be obtained from 89 participants (44 ASC, 45 CMP). The functional connectome was calculated separately for all acquisitions and was then averaged over acquisition to generate one functional connectome per participant. To reduce the influence of motion on the functional connectivity results [35], we employed a combination of approaches. First, we assessed raw data quality using a comprehensive set of quality indicators [36]. Second, we employed regression of noise and motion parameters [37], bandpass-filtering (0.009–0.1 Hz), and spatially smoothing (3 mm full-width-half-maximum). Third, we controlled for image quality in the statistical analysis (see Additional file 1). These procedures were carried out using *nilearn* v0.50 [38].

The functional connectome shows modular structure that is highly similar to the large-scale functional networks that are identified through other methods and that are found to be active during particular tasks [35]. To determine the module structure, we employed consensus community detection [39], an optimization clustering approach based on the Louvain method [40], and tuned the resolution of the clustering using a mutual information criterion (see Additional file 1 for a detailed description).

### Graph theory analysis

Graph theory measures were used to characterize the functional brain networks and compare them across participants. All graph analyses were based on weighted networks after applying the optimal density threshold. The purpose of thresholding is to remove the influence of weak connections that may be spurious [41]. Here, we applied the method described in a previous study on aging [37]. This method tunes the optimal threshold by optimizing the information that can be obtained at the group level. After thresholding, the results of graph theory analysis may be influenced by unconnected nodes. We carried out an additional analysis that just focused on the largest connected component in each functional connectome. The results were consistent with the findings based on the thresholded functional connectomes. The detailed analysis is presented in the Additional file 1.

The functional brain network shows a small-world topology, characterized by regional clustering and a short average path length (high efficiency) [18]. This organization is thought to maximize regional communication and retain efficient global communication. In addition, the human brain exhibits a modular structure with a few highly connected hub nodes that are thought to be central for information transfer [42, 43]. To characterize the organization of the functional brain network in the current analysis, we focused on three graph metrics, i.e., the average clustering coefficient, *C*_*G*_, global efficiency, *E*_*G*_, and the participation coefficient *P*_*G*_. The global clustering coefficient is an index of clustering within a graph. Global efficiency is the inverse of path length and indicates the ease of transfer within a graph. Global measures for both metrics are calculated by averaging across all nodes within the network. Further, to characterize the modular organization of the functional connectomes, module-level metrics of local efficiency, *E*_*g*_, and participation coefficient, *P*_*g*_, were calculated. *E*_*g*_ is the inverse of the shortest path length of nodes within a given module *g*. *P*_*g*_ indicates the diversity of intermodular connections within a given module *g*. The graph theory measures were calculated as described by Rubinov and Sporns [44]. Because the data-driven modularity solution did not distinguish some of the canonical functional networks, additional analyses were carried out with the modularity solution presented in Power et al. 2011.

### Default mode network connectivity

Differences in the default mode network (DMN) are consistently identified in ASC. Further, age-related changes in DMN connectivity have been documented in ASC [10–12] and in typical groups [45, 46]. Further, specific connections within the DMN may be increased in ASC, i.e., connection between the posterior cingulate cortex (PCC) and the parietal cortex, while other are decreased [14]. Because of the central importance of the DMN in ASC indicated by the literature, we focused specifically on connections within the DMN in additional analyses.

To obtain connection strength between DMN regions, the averaged blood oxygen level dependent signal (BOLD) signal within 5 mm spheres was calculated for ROIs placed in the PCC (MNI: − 2, − 36, 18), medial prefrontal cortex (MNI: 0, 52, − 6), and left and right parietal cortex (MNI [left]: − 48, − 62, 36; MNI [right]: 46, − 62, 32). The ROI definitions were based on a seminal large-N study that established the reliability of DMN functional connectivity [47]. Subsequently, the partial correlation between signals of all binary combinations of these ROIs was calculated controlling for signal in the other ROIs. We employ partial correlations here to disambiguate specific connections within the DMN from general connectivity patterns.

### Cortical morphology

Age- or group-related differences in cortical morphology may influence functional connectivity estimates. Our previous analysis of cortical morphology in the same sample as the current analysis indicated significant age-related differences across the ASC and CMP groups [20]. To ascertain that differences in functional connectivity were not due to differences in cortical morphology, we present additional statistical models that include morphology (intracranial volume, cortical thickness, cortical surface area) as regressors following studies on aging [37, 48]. To this end, global measures (cortical thickness, surface area) were extracted from the FreeSurfer summary statistics. For local measures of cortical morphological, maps of cortical thickness and cortical surface area were extracted via FreeSurfer and transformed from a surface- to a volume-representation. Subsequently, the morphology maps were transformed to MNI152 space and morphology values were extracted by averaging values within a 5-mm radius around the ROI coordinates. A detailed analysis of differences in cortical morphology in this sample is presented in [20].

### Statistical analysis

For the statistical analysis of the association between age, group, and their interaction with graph measures, we employed a non-parametric permutation-regression analysis. A multiple regression model was fitted with the graph theory measure as the outcome and predictors of age, participant group, and their interaction. The summed functional connection strength was included as a nuisance regressor to account for non-specific differences in connectivity [49]. There was no significant difference between the groups in summed connection strength (ASD: mean = 6411.35, SE = 394.429; CMP: mean = 6043.71, SE = 327.247; Welch-corrected *t* test: *t* (83.79) = 0.72, *p* = 0.475). In addition, the AFNI image quality index (aqi) was included as a nuisance regressor because this index showed a significant association with age (see Additional file 1). Further, functional connectivity may be influenced by medication [50]. Therefore, we ran additional models with psychotropic medication as a regressor.^2^ For each model, the outcome variable was randomly shuffled 10,000 times to obtain a null distribution of regression coefficients. Then, the observed regression coefficient was compared to this distribution. Further, the confidence interval for each regression coefficient was obtained by randomly selecting 80% of the data across the 10,000 permutations. Bonferroni-correction was applied to account for multiple comparisons across modules and the corrected *p* values are reported. For the comparison of connection strength within and between modules, false discovery rate (FDR) correction using the Benjamini-Hochberg procedure was applied.

### Behavioral tasks

To relate functional differences to behavior, we investigated the association between functional brain organization and performance for two out-of-scanner tasks. Participants were assessed on a Flanker task [51] that has been shown to be challenging for both individuals with ASC and older adults [52, 53]. Following our previous analysis of the behavioral data [19], we focused on intra-individual variation of reaction time (IIVRT) which is a sensitive measure of cognitive aging [54, 55]. Two measures were used to characterize IIVRT, i.e., the standard deviation of reaction time (sdRT) and mean reaction time (MRT) variation (CV = sdRT/MRT). For statistical analysis, the partial correlation between sdRT or CV with graph measures controlling for image quality (aqi) were calculated and transformed using Fisher’s r-to-z. Subsequently, *z* values were compared between the groups (see Additional file 1 for details).

Individuals with an ASC often have difficulties with processing social information and this has been related to functional connection strength, particularly within the DMN [56, 57]. Further, social processing ability has been found to decline in typical aging [58]. To assess social processing, a verbal Faux-pas task [59] was administered. The number of correctly identified faux-pas relative to the number of correctly answered factual questions was used for the analysis.

## Results

### Functional networks

**Fig. 1:**
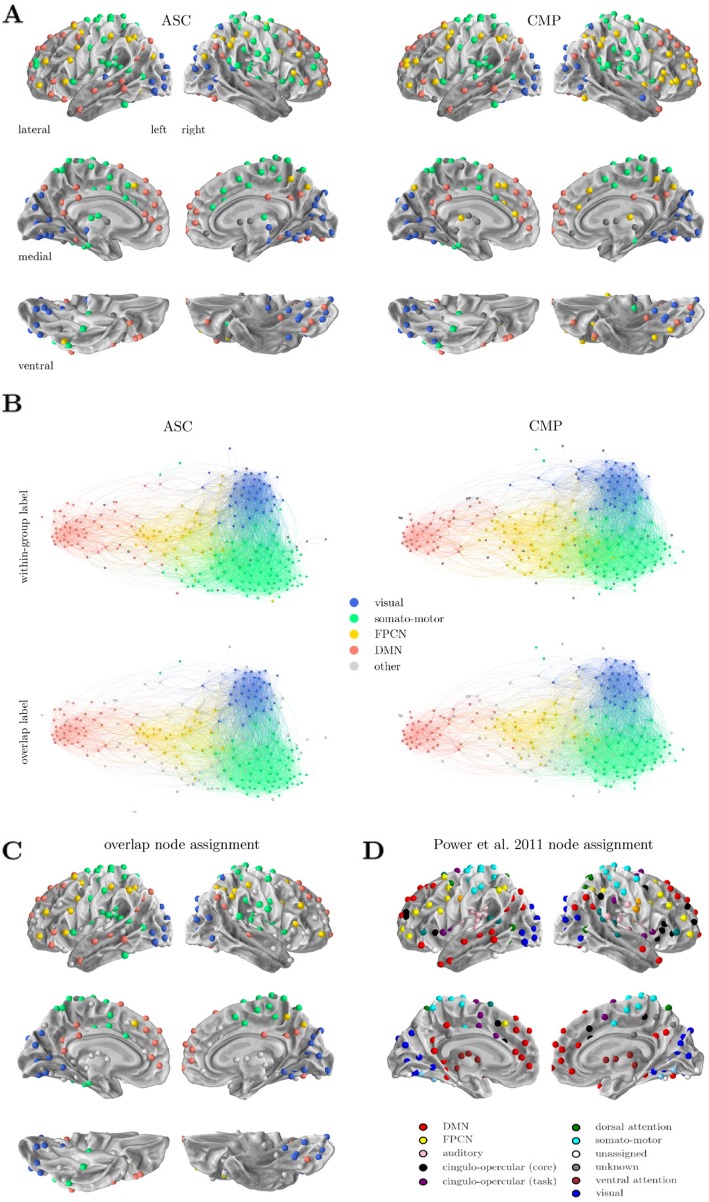
**a** Illustration of the functional modules identified in the ASC group (left) and in the CMP group (right). **b** Presentation of the functional networks in Force Atlas layout [60]. Only positive connections are shown for the purpose of this illustration. The top figures show the network with the module assignment identified within each group. The bottom figures show the module assignment of nodes that were assigned to the same network in the ASC and CMP group. **c** Final assignment of nodes to modules based on the overlap between both groups. **d** Node assignment according to the modularity solution presented in Power et al. 2011

Functional modules were identified using consensus community detection alongside a tuning procedure to identify the optimal community resolution [37]. There was no significant effect of age, group, or their interaction on the modularity index (age: β = − 0.18, (− 0.03, − 0.32) [median, (5%ile, 95%ile)], *p* = 0.237; group: β = − 0.05, (− 0.23, 0.14), *p* = 0.835; age × group: β = − 0.10, (− 0.31, 0.09), *p* = 0.663). The optimal community resolution for the ASC group was identified at γ = 1.3 and a modularity index of 0.61 (SE = 0.015, range 0.34–0.77; see Fig. 1a). The optimal solution for the CMP group was found at γ = 2.7 and at a modularity index of 0.60 (SE = 0.015, range = 0.27–0.76). In both groups, the identified modules were similar to modules previously described for typical adults [26], specifically the canonical visual, frontoparietal control (FPCN), and default mode network (DMN) could be clearly identified. In contrast to previous results, there was no distinction between the somatomotor network and the insula nodes of the cingulo-operacular networks in either group. These nodes were assigned to one network, labeled the somatomotor network here. The module solution for the CMP group contained two additional modules, namely a hand region subgraph of the somatomotor network and higher-level visual network. For consistency across the groups, these additional modules were subsumed in the somatomotor and visual network respectively. Using this assignment, there was good agreement with 189 out of 237 nodes being assigned to the same module across both groups (79.75% overlap, see Table 2 and Fig. 1b). These common labels were used to create an overlap module solution for further analysis (see Fig. 1c). For reference, the original labels reported by Power et al. [26] are shown in Fig. 1d.

**Table 2:** Overview of agreement between modules identified in the ASC and CMP group

ASD							
	Visual	Somatomotor	FPCN	DMN	Other	Total	
CMP							
Visual	35	4	0	0	4	43	81%
Somatomotor	0	62	15	0	5	82	76%
FPCN	2	0	29	2	1	34	85%
DMN	2	1	5	42	6	56	75%
Other	1	0	0	0	21	22	95%
Total	40	67	49	44	37	237	
% in common	88%	93%	59%	95%	57%		

### Differences in graph metrics

**Fig. 2:**
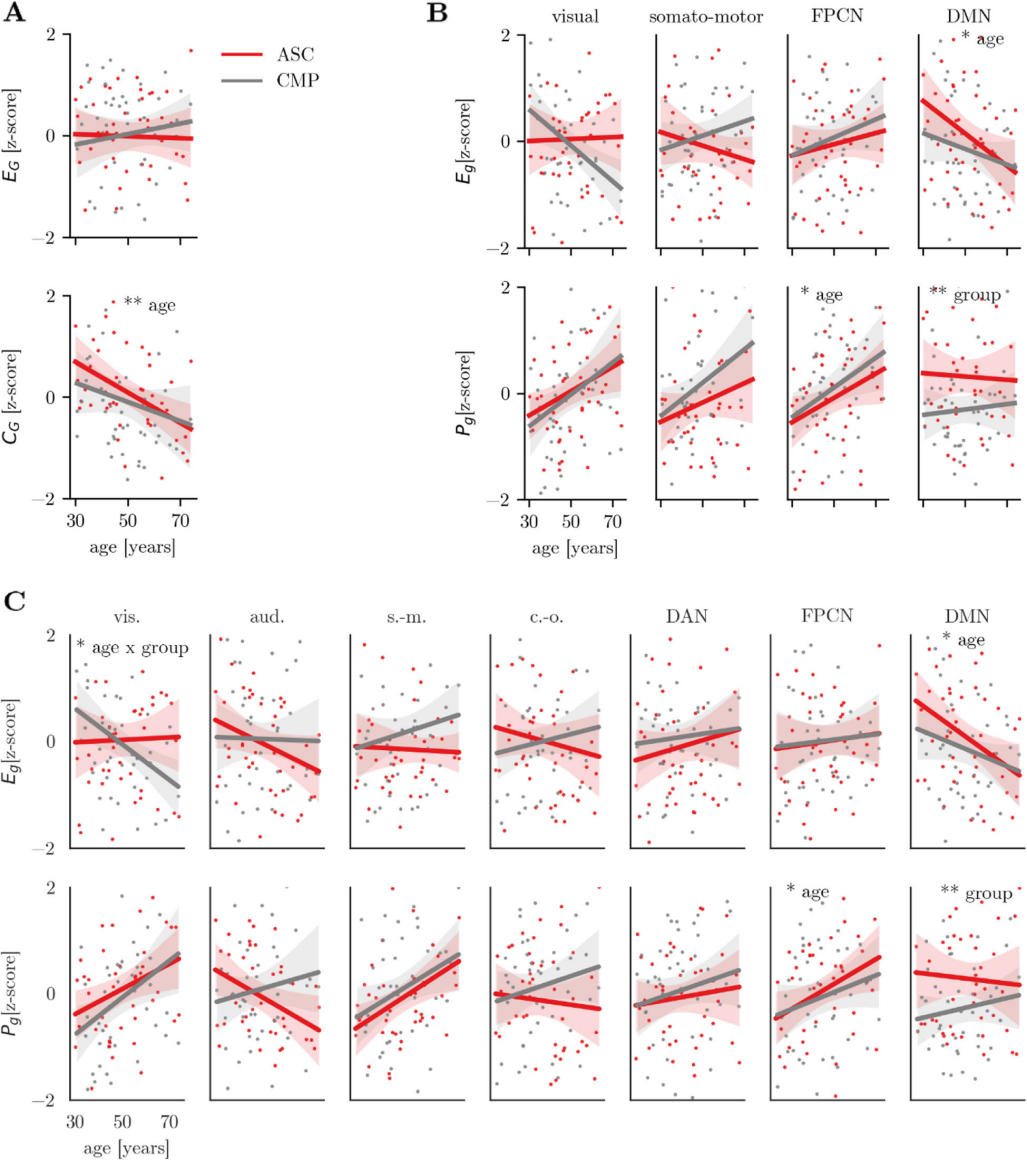
Overview of graph theory results. **a** Global graph theory metrics global efficiency (*E*_G_) and average clustering coefficient (*C*_G_). **b** Module-level graph metrics local efficiency (*E*_g_) and participation coefficient (*P*_g_) for the module solution identified in the current sample. **c** Module-level graph metrics for the major networks in the modularity solution presented in Power et al. 2011. For all the figures, the residuals are shown after regressing the effect of image quality (aqi) and total connection strength. The shaded area around the regression line shows the 5–95%ile confidence interval based on a bootstrap sample with 5000 permutations. Legend: **p < 0.01, *p < 0.05. *vis*. visual, *aud*. auditory, *s*.-*m*. somatomotor, *c*.-*o*. cingulo-opercular, *DAN* dorsal attention network, *FPCN* frontoparietal control network, *DMN* default mode network

Regarding brain-wide global graph metrics, statistical analysis indicated a significant effect of age for the global clustering coefficient *C*_*G*_ with older age being associated with lower *C*_*G*_ (see Fig. 2a, age: β = − 0.36, (− 0.47, − 0.28) [median, (5%ile, 95%ile)], *p* = 0.0122). This effect was robust to the inclusion of psychotropic medication use as a regressor (age: β = − 0.36, (− 0.47, − 0.28), *p* = 0.0132) but was no longer significant when controlling for whole-brain cortical thickness, cortical surface area, and intracranial volume (age: β = − 0.32, (− 0.49, − 0.19), *p* = 0.097). There was no significant association for global efficiency *E*_*G*_.

For module-level graph measures, statistical analysis indicated a significant effect of decreasing *E*_*DMN*_ with age (see Fig. 2b, β = − 0.36 (− 0.48, − 0.24), *p* = 0.015). Further, older age was associated with a higher *P*_*FPCN*_ (β = 0.32 (0.21, 0.42), *p* = 0.031). A significant group difference was indicated for *P*_*DMN*_ with lower *P*_*DMN*_ in the CMP group compared to the ASC group (β = − 0.63 (− 0.82, − 0.47), *p* = 0.003). These effects remained when controlling for regional cortical thickness and cortical surface area (*E*_*DMN*_ - age: β = − 0.36 (− 0.51, − 0.24), *p* = 0.013; *P*_*DMN*_ - group: β = − 0.63 (− 0.81, − 0.47), *p* = 0.003). The association between age and *E*_*DMN*_ was also indicated when controlling for psychotropic medication use (*E*_*DMN*_*-age*: β = − 0.36 (− 0.50, − 0.24), *p* = 0.016), but the group differences in *P*_*DMN*_ was no longer significant (*P*_*DMN*_ - group: β = − 0.31 (− 0.57, − 0.09), *p* = 0.226). The association between age and *P*_*FPCN*_ was no longer significant when controlling for regional morphology (β = 0.28 (0.16, 0.37), *p* = 0.068) or psychotropic medication use (β = 0.28 (0.18, 0.39), *p* = 0.059). Using the Power et al. 2011 module solution, the results indicated an additional age × group interaction for *E*_*Visual*_ whereby the CMP group showed lower *E*_*Visual*_ with age while there was no age-related difference in the ASC group (see Fig. 2c, age × group: β = − 0.41 (− 0.63, − 0.22), *p* = 0.038). This effect was no longer significant when controlling for cortical morphology (β = − 0.41 (− 0.62, − 0.22), *p* = 0.061) or psychotropic medication (β = − 0.41 (− 0.61, − 0.17), *p* = 0.071). The other findings matched the results obtained with the data-driven modularity solution. There were no other significant effects of age, participant group, or the age × group interaction for *E*_*g*_ or *P*_*g*_ for any other module in either modularity solution. See Fig. 2d for a graphical overview of the results. See Table 3 for an overview of the results.

**Table 3:**
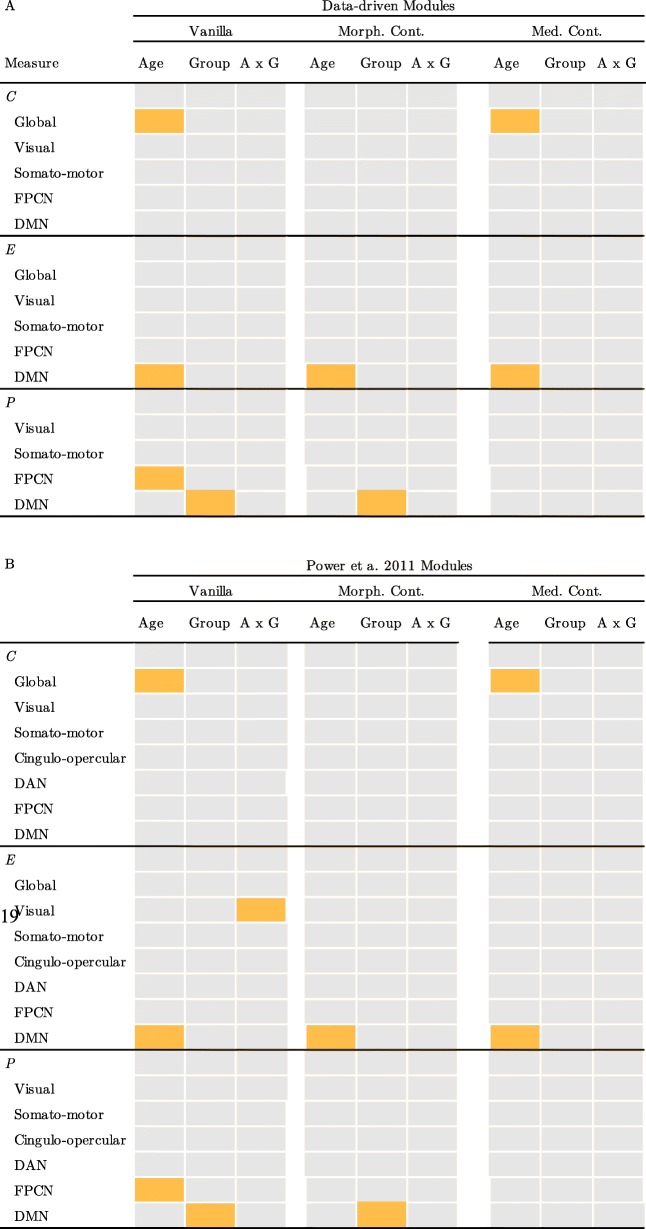
Overview of the graph theory results. A: Results using the data-driven module solution B: Results using the Power et al. 2011 solution

Schematic overview of the results. Orange squares indicate significant effects after controlling for multiple comparisons across modules. Gray squares indicate that no significant effect was found. *C* clustering coefficient, *E* efficiency, *P* participation coefficient, Vanilla: model that does not include brain volume and cortical morphology, Morph. Cont.: model that does include brain volume and cortical morphology as regressors, Med. Cont.: model that includes medication use as a regressor

Analysis of the association between IIVRT and module-level graph metrics indicated a significant difference for *P*_visual_ between the groups. The ASC group showed a negative correlation between *P*_visual_ and sdRT, while the CMP group showed a positive correlation (ASC: *r* = − 0.31, CMP: *r* = 0.26, Δz = − 2.54, *p* = 0.044). There were no significant effects of age, group, or their interaction for the Faux-pas task (all *p* > 0.2).

### Differences in module connection strength

**Fig. 3:**
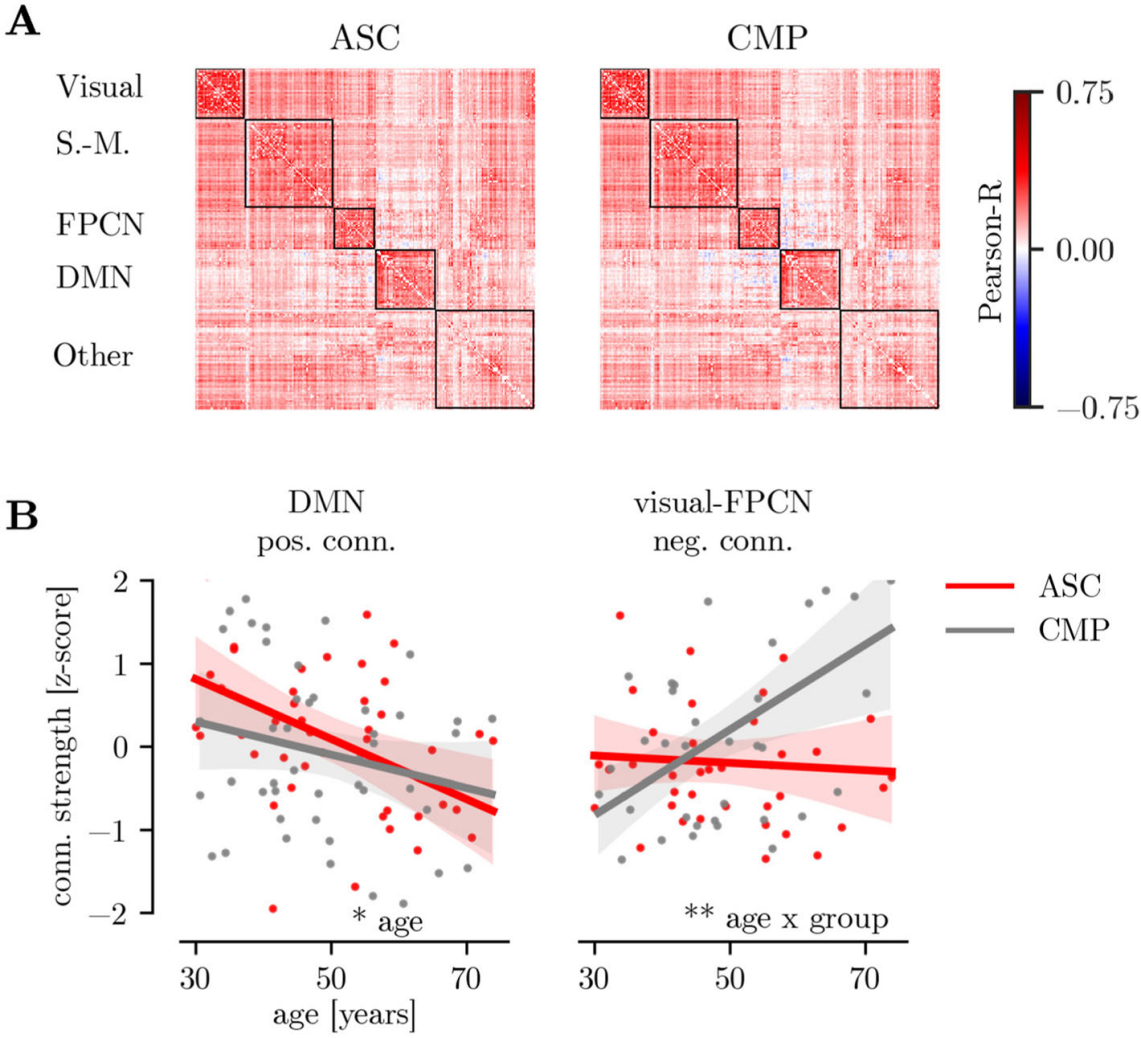
Differences in connection strength. **a** Average connection matrices for the ASC and CMP group ordered according to the data-driven module solution. The outlines indicate the module boundaries. **b** Summed positive connection strength within the DMN and summed negative connections strength of connections between the visual network and the FPCN. The shaded areas indicate the 5–95%ile confidence interval for each group based on a bootstrap sample with 5000 repetitions. Legend: **p < 0.01, *p < 0.05

Differences in connection strength associated with age and ASC status were evaluated to assess general shifts in functional connectivity patterns. Within-network connection strength was negatively associated with age (age: β = − 0.29 (− 0.47, − 0.17) [median, (5%ile, 95%ile)], *p* = 0.003). There were no effects of age, group, or their interaction for between-module connection strength (all *p* > 0.3). Analysis of positive and negative connection strength for individual modules indicated that positive DMN connection strength declined with age (see Fig. 3b, age: β = − 0.38 (− 0.49, − 0.26), *p* = 0.011). This effect remained when controlling for cortical thickness and cortical surface area (age: β = − 0.47 (− 0.61, − 0.29), *p* = 0.005), but not when controlling for psychotropic medication (β = 0.09 (− 0.08, 0.25), *p* = 0.671). Further, there was an age × group interaction for negative connections between the visual network and the FPCN (age × group: β = 0.75 (0.56, 0.91), *p* = 0.006). Visual-FPCN connections became less negative with age in the CMP group, but showed no association with age in the ASC group (see Fig. 3b). This effect remained when controlling for differences in cortical morphology (age × group: β = 0.67 (0.49, 0.84), *p* = 0.003), but not when controlling for the effect of psychotropic medication (β = 0.22 (0.01, 0.41), *p* = 0.286).

The analysis of connection strength using the Power et al. 2011 parcellation indicated an additional significant negative association between age and within-module connection strength of the cingulo-opercular network (β = − 0.33 (− 0.43, − 0.22), *p* = 0.023). This effect was robust to the inclusion of psychotropic medication use (β = − 0.33 (− 0.43, − 0.22), *p* = 0.023), but was not significant when controlling for cortical morphology (β = − 0.26 (− 0.36, − 0.15), *p* = 0.081). There were no other significant effects of age, group, or their interaction for either parcellation.

Regarding the relationship with behavioral performance, the analysis of the association between connection strength and IIVRT indicated no significant difference between the groups for any positive or negative within- or between-module connection (all *p* > 0.1). There were no significant effects of age, group, or their interaction for the Faux-pas task (all *p* > 0.1).

### Differences in DMN connection strength

**Fig. 4:**
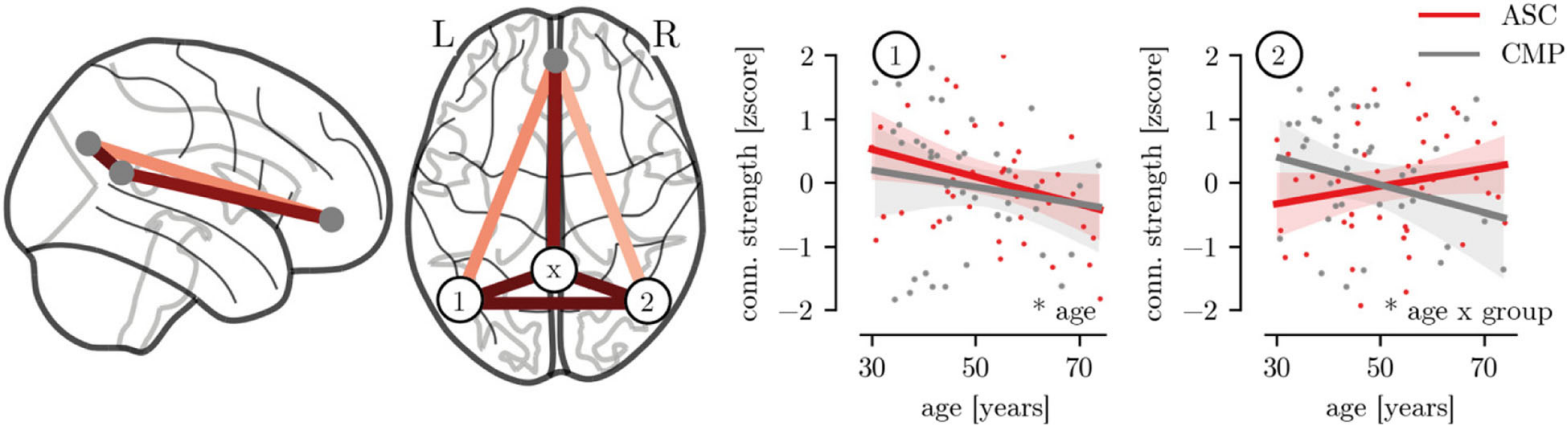
Connection strength within the default mode network (DMN). The left panel illustrates the connections of the DMN included in the analysis. The color indicates the relative connection strength. (1) Left temporo-parietal junction (TPJ), (2) right TPJ, (x) posterior cingulate cortex (PCC). The right panels show the relationship between the connection strength, age, and group. Legend: *p < 0.05

Analysis of DMN connections indicated a significant reduction in the strength of the connection between the left temporoparietal junction (TPJ) and the posterior cingulate cortex with age (see Fig. 4, age: β = − 0.29 (− 0.47, − 0.17) [median, (5%ile, 95%ile)], *p* = 0.043). This effect was no longer significant when controlling for differences in cortical thickness and surface area (β = − 0.28 (− 0.49, − 0.13), *p* = 0.065) or psychotropic medication (β = − 0.27 (− 0.44, − 0.15), *p* = 0.057). Further, a group × age interaction was indicated for the connection between the right TPJ and the PCC with an age-related reduction in connection strength for the CMP group but not for the ASC group (age × group: β = − 0.48 (− 0.66, − 0.28), *p* = 0.027), which remained when controlling for cortical morphology (β = − 0.49 (− 0.68, − 0.29), *p* = 0.022) or psychotropic medication use (β = − 0.49 (− 0.68, − 0.31), *p* = 0.019).

Regarding the relationship with the behavioral measures, there were no significant differences between the groups in the association of IIVRT and connection strength for any DMN connection (all *p* > 0.1) or any significant effects of age, group, or their interaction for the Faux-pas task (all *p* > 0.1).

## Discussion

The current study investigated age-related differences in functional brain organization in autistic adults of middle and older age. The results indicated both parallel and divergent brain aging in autistic adults.

On one hand, this study indicated age-related reductions in the connection strength and local efficiency of the DMN in both the ASC and the comparison group. Several studies of typical aging indicate that DMN connectivity is a sensitive and robust marker of brain age [37, 61–63]. In addition, several risk factors for dementia have been found to relate to DMN activity [48, 64–67]. Based on the current study, we found no indication of age-related differences in DMN connectivity in ASC as compared to typical aging consistent with a parallel aging account.

Further, the current study suggested that reduced integration of the DMN is a stable feature of ASC across middle and older age. This result mirrors similar findings in young people with an ASC. For instance, Nomi et al. reported an age-related pattern of hyper-connectivity of the DMN in childhood. Yet, no such differences were found in middle adulthood in the same study [68]. In light of these findings, the current results suggest that hyper-connectivity of the DMN may recur in middle and older age in ASC. Alternatively, age-related decline in DMN integration may already be present at middle age in typical aging [69]. Extrapolating from these findings, the higher participation coefficient in the ASC group could potentially reflect a more “youthful” connectivity pattern. However, this is currently only speculative and further studies that span childhood and older age are needed to distinguish these alternative accounts and establish if higher DMN integration is a protective or risk factor for aging in ASC. In summary, the results of this study suggest that a higher participation coefficient of the DMN is a stable feature of ASC across middle and older age.

In addition to the DMN-related differences, our results indicated an age-related increase in the participation coefficient of the frontoparietal control network in the ASC and CMP group. This finding is in line with the general age-related decrease in network segregation across functional networks [70] and specific age-related associations for the FPCN [71, 72]. The FPCN may play a central role in brain aging due to its role in controlling other functional networks [73], particularly the DMN [74]. Further, FPCN integration may be particularly important for ASC. The only published study on functional brain aging in ASC found a significant reduction in FPCN connection strength in a small sample of middle-aged adults with an ASC that related to impaired social processing. The greater integration of the FPCN indicated in the current study may reflect part of a compensatory mechanisms as has been suggested in typical aging [73, 75].

We also observed some divergent patterns of age-related changes between the ASC and CMP group. Specifically, the CMP group showed an age-related reduction in the connectivity of the visual network. In contrast, there was no age-related change in the ASC group. Further, negative connections between the visual network and FPCN that became less negative with age in the control group did not change in the ASC group. The observed associations may indicate dedifferentiation by which the visual network becomes less segregated and shows less decoupling with the FPCN in typical aging. Similar age-related reductions in sensory networks and their de-segregation from cognitive networks have been observed in studies of typical aging [76, 77]. In fact, degradation in sensory processing commonly precedes and later aggravates cognitive problems in typical aging [78]. Notably, behavioral studies indicated that older individuals with ASC seem to show less age-related differences in visual memory but not verbal memory [4, 15]. The reduced age-related differences in visual network function in ASC may be neural substrates of the persevered visual memory function. The different association with reaction time variability in ASC indicated by the current study may suggest that the differences in visual network integration are meaningful for cognitive performance. In brief, visual network integration appears stable across middle and older age in ASC but may show age-related decline in typical aging.

Contrary to our expectations, we did not find an effect of ASC status or age for the relationship between performance on the Faux-pas task and any functional connectivity measure. There are several potential reasons for these negative findings. First, the number of older participants (> 67 years [79]) may have been too small to detect the decline in social processing associated with typical aging. Second, older autistic adults may no longer show difficulties on social processing tasks [4], despite continuing challenges with social functioning [80]. Future studies with dedicated task assessments, e.g., [81], will be needed to firmly establish the link between brain function and social processing in older autistic adults.

It is important to keep in mind some limitations of this study. First, the study was cross-sectional. Consequently, the associations that were identified may be confounded with differences between age cohorts. Further, all included adults had received their official ASC diagnosis during adulthood—note that the participants were over 10 years old when autism was introduced in the DSM-III [82]. Several steps were taken to ensure a valid diagnosis (see [20] for a detailed discussion), but a late diagnosis may still imply that we included a sample with relatively mild ASC symptomatology. A further limitation is the difference in medication between the ASC and CMP groups in the current study. Psychotropic medication exposure is an important potential confound in adult ASD research given that such medication is commonly prescribed [83] and is known to influence brain functional connectivity [50]. We aimed to investigate the influence of psychotropic medication in additional regression models. However, the current analysis could not distinguish between type of medication, dosage, and duration of treatment that may affect connectivity differently. In addition, the control analyses of medication may introduce additional confounds because psychotropic medication use was more prevalent in the ASC group and because individuals with more age-related complaints are more likely to be treated with medication. These limitations will need to be addressed in future studies based on broader samples.

Another limitation is that the current study did not assess cardiovascular health that may show differences in older age and in ASC that may affect the fMRI BOLD signal [84, 85]. Future studies should include parallel heart rate recording and corroborate findings with other imaging modalities, e.g., M/EEG, PET. Moreover, many of the aging effects in functional connectivity may reflect early stages of dementia that are not apparent in cognitive assessments. Future studies of aging in ASC should employ a broader set of sensitive cognitive measures [86] and potentially incorporate biomarkers [87]. Furthermore, the current study cannot distinguish between direct effect of ASC and effects that arise from differences in life experience that are associated with ASC, e.g., see [88]. Future studies that assess lifestyle differences, ideally in a longitudinally sample, will need to disentangle these effects.

In conclusion, the current study finds support for both parallel and divergent aging in ASC in middle and older age. Similar selective differences in some aspects of aging alongside parallel aging in other indicators have been reported in schizophrenia and ADHD [16, 89, 90]. Specifically, the current study found that age-related negative associations in the connectivity of the default mode network and diminishing segregation of the frontoparietal control network with age were found to be similar in ASC. In addition, the current results indicated reduced age-related negative association in the visual network in ASC that showed a different relation with reaction time variability in ASC. A potential interpretation of this finding is that the biological processes associated with ASC protect against age-related decline in functional connectivity of the visual network. In sum, the results highlight the complexity of brain organization in ASC with similarities and differences to CMP groups across different segments of the lifespan.

## Supplementary information

**Additional file 1.** The additional file contains further details regarding the methodological approach and presents results of control analyses.

## Data Availability

The datasets used during the current study are available from the corresponding author on reasonable request. The code for the analysis is available on the Open Science Framework website: https://osf.io/kvz6q/?view_only=b466e6eb837048b48b7d65f3965ab4db

## Abbreviations

ADOS: Autism Diagnostic Observation Schedule
AQ: Autism spectrum quotient
aqi: AFNI image quality index
ASC: Autism spectrum condition
BOLD: Blood oxygen level dependent signal
*C*_*G*_: Global clustering coefficient
*C*_*g*_: Local clustering coefficient for node *g*
CMP: Comparison group
DMN: Default mode network
*E*_*G*_: Global efficient
*E*_*g*_: Local efficiency for node *g*
FPCN: Fronto-parietal control network
IIVRT: Intra-individual variation of reaction time
MNI152: Montreal Neurological Institute stereotaxic space
PCC: Posterior cingulate cortex
*P*_*g*_: Participation coefficient for node *g*
ROI: Region of interest
rsfMRI: Resting-state functional MRI
SE: Standard error
TPJ: Temporoparietal junction

## Footnotes

1 We use the term of “autism spectrum condition” throughout this manuscript because the traditionally used term “autism spectrum disorder” carries unjustified negative connotations. Further, we refer to adults with an ASC diagnosis as ‘autistic adults’ because recent research indicates that identity-first language (as opposed to person-first language) is the terminology that adults themselves prefer [1].

2 We summarized all psychotropic medication in a dummy variable with 0 for “no psychotropic medication” and 1 for “takes psychotropic medication.” The effect of specific medication could not be assessed, because the medication prescribed to participants in the sample was varied with only few participants taking the same medication. Both the ASC and CMP group were included in this analysis. Please note that psychotropic medication use was more common in the ASC group (see Table 1).
