# Supplementary material for: Age-variant and age-invariant features of functional brain organization in middle-aged and older autistic adults
Source: Mol Autism. 2020 Jan 22;11:9. doi: 10.1186/s13229-020-0316-y (PMC6977283; doi:10.1186/s13229-020-0316-y)
Supplement: Supplementary file 1 — Additional file 1. The additional file contains further details regarding the methodological approach and presents results of control analyses. [file 13229_2020_316_MOESM1_ESM.pdf]

Supplementary Methods

Reproducible code for all analyses presented in the main manuscript and the supplementary materials is available on the Open Science Framework website:

fMRI sequences and tasks

The functional MRI acquisition protocol consisted of two task-related and one resting-state sequence. The basic scan parameters were identical for all sequences: Repetition time (TR): 2s, Echo time (TE): 27.63ms, Flip angle: 76.1°, Voxel size: 3x3x3.3 mm^3. For the *resting-state*, participants were instructed to close their eyes and let their mind wander. The resting sequence lasted 5 minutes. The first task was designed to tap *inhibitory control*. Participants had to rapidly respond to target stimuli while distractor stimuli were presented. An adapted Flanker task was used that consisted of distractor stimuli that were either congruent or incongruent at the stimulus identification or response selection level. The Flanker task took 9.94 min. The second task was a *social processing* task. Participants had to distinguish between faces and scrambled faces, i.e. Mooney faces (16). The same paradigm and stimuli as described by Grützner et al. 2010 were used (15). The social processing task took 7.1 min.

MRI pre-processing

All images were converted from native PAR/REC to NIfTI-1 format using the dicm2nii toolbox (https://github.com/xiangruili/dicm2nii). The T1-weighted (T1w) image was corrected for intensity non-uniformity (INU) using *N4BiasFieldCorrection* (1)(ANTs 2.2.0), and used as T1w-reference throughout the workflow. The T1w-reference was then skull-stripped using *antsBrainExtraction.sh* (ANTs 2.2.0), using OASIS as target template. Brain surfaces were reconstructed using recon-all (2)(FreeSurfer 6.0.1, RRID:SCR_001847), and the brain mask estimated previously was refined with a custom variation of the method to reconcile ANTs-derived and FreeSurfer-derived segmentations of the cortical grey-matter of Mindboggle (3)(RRID:SCR_002438). Spatial normalization to the ICBM 152 Nonlinear Asymmetrical template version 2009c (4)(RRID:SCR_008796) was performed through nonlinear registration with *antsRegistration* (5)(ANTs 2.2.0, RRID:SCR_004757), using brain-extracted versions of both T1w volume and template. Brain tissue segmentation of cerebrospinal fluid (CSF), white-matter (WM) and grey-matter (GM) was performed on the brain-extracted T1w using *FAST* (6)(FSL 5.0.9, RRID:SCR_002823).

For each of the 3 BOLD runs per subject (across all tasks and sessions), the following preprocessing was performed. First, a reference volume and its skull-stripped version were generated using a custom methodology of *fMRIPrep*. The BOLD reference was then co-registered to the T1w reference using *bbregister* (FreeSurfer) which implements boundary-based registration (7). Co-registration was configured with nine degrees of freedom to account for distortions remaining in the BOLD reference. Head-motion parameters with respect to the BOLD reference (transformation matrices, and six corresponding rotation and translation parameters) are estimated before any spatiotemporal filtering using *mcflirt* (8)(FSL 5.0.9). The BOLD time-series were resampled onto their original, native space by applying a single, composite transform to correct for head-motion and susceptibility distortions. These resampled BOLD time-series will be referred to as *preprocessed BOLD*. The BOLD time-series were resampled to MNI152NLin2009cAsym standard space, generating a *preprocessed BOLD run in MNI152NLin2009cAsym space*. First, a reference volume and its skull-stripped version were generated using a custom methodology of *fMRIPrep*. Several confounding time-series were calculated based on the *preprocessed BOLD*: framewise displacement (FD), DVARS and three region-wise global signals. FD and DVARS were calculated for each functional run, both using

their implementations in *Nipype* (following the definitions by (9)). Global signals were extracted within a CSF and a WM mask defined in the anatomical image for confound regression.

Susceptibility artefact of ROIs

Due to susceptibility artefacts some ROIs contained insufficient signal to estimate their functional connectivity reliably. To remove ROIs that were affected by susceptibility artefacts, brain masks were created for each participant by thresholding the average BOLD image at 70% intensity(10). The 27 ROIs that showed <50% overlap with this mask in one or more participants were excluded from the analysis.

Quality control

Scans with measures of image artefacts (standardised framewise displacement [DVARS], AFNI's quality index, AFNI's outlier ratio) that fell two standard deviations above the mean were excluded from further analysis. This resulted in the exclusion of 32 scans. There was no significant difference in quality metrics between groups after removing these scans (all $p>0.3$). There was a significant linear relationship between age and one image quality metric (Simple linear regression: aor: $\beta=0.09$, $p=0.171$, aqi: $\beta=0.17$, $p=0.011$, dvars: $\beta=-0.05$, $p=0.436$). The image quality index (aqi) was therefore included as a covariate in subsequent analyses. Further, we regressed the effect of motion parameters and of signals within the CSF and WM masks. This included the extracted time courses, their squared term, first temporal derivative, and squared derivative (28) resulting in a total of 32 regressors. In addition, a bandpass filter was applied between 0.009 and 0.1Hz and the data were spatially smoothed with a Gaussian kernel with a 3mm radius (FWHM).

Determining the optimal density threshold for community detection

Typically, a threshold is applied to the connectivity matrices in order to remove the influence of weak connections that may be false positives. In the current analysis, the threshold was determined as the density that showed the best consistency of connections across participants, defined as the lowest entropy of the binarized connection matrix across participants normalized by the same metric calculated over 50 scrambled versions of the matrices (11). The lowest entropy was found at 1% density for the ASC group and at 1.1% density for the CMP group. To use a consistent threshold for community detection, the middle value of 1.05% density was used.

Consensus community clustering

The consensus community clustering approach based on the Louvain method (12), which creates a module partition by maximising the number of within-module connections while minimizing the number of between-module connections. Correlation matrices were submitted to the community detection algorithm after removing connections between regions in close spatial proximity (<20mm, (10)) and thresholding the networks to a target density. Subsequently, the community solution was fine-tuned using the Kernighan-Lin algorithm (13). The community detection relies on initial random assignment of nodes to communities and may, therefore, vary between runs. In order to reach a stable solution, the community detection was repeated 50 times to create an agreement matrix which was used to create a consensus community assignment (10). The community solution across all participants was used to create a group-level community solution using the same consensus procedure. The entire process was repeated across different resolutions by varying the gamma parameter of the Louvain algorithm

(range: 1.0 to 3.0 with 0.1 increments). The optimal resolution was determined as the resolution that showed the highest normalised mutual information between subsequent values of gamma.

Statistical analysis of the IIVRT data

Group comparison of z-values: $\Delta z = (z_{ASD} - z_{CMP})/\sqrt{(\frac{1}{N_{ASD}-3} + \frac{1}{N_{CMP}-3})}$

Statistical comparison between the groups with reference to the cumulative distribution function (*normcdf*): $p = (1 - normcdf(abs(\Delta z))) * 2$

Supplementary Results

Graph analysis using the largest connected component

Graph theory metrics may be influences by unconnected nodes. Because the threshold was tuned using the optimal density threshold, we could not ensure that the resulting graphs would be full connected. To evaluate if this influenced the results, we compared the size of the largest component between groups. There was no statistically significant difference between the ASD and CMP group (ASD: mean=119.45, SE=2.459; CMP: mean=118.21, SE=2.882; Welch-corrected t-test: t(83.93)=0.33, *p*=0.742). Further, we repeated the graph theory analysis with only the largest connected component in each network. The results were consistent with the results reported for the full functional connectome (see Figure S1). As in the main analysis, there was a significant association between age and C_G (β=-0.37 (-0.47, -0.28), *p*=0.013, model controlling for image quality [aqi] and total connectivity). For module-level metrics, there was a negative association between age and E_{DMN} (β=-37, (-0.51, -0.24), *p*=0.011) as indicated in the analysis of the full connectome. Further, there was a significant effect of group for the P_{DMN} (β=-0.63 (-0.81, -0.47), *p*=0.029) consistent with the results in the main analysis. In contrast to the main analysis, the association between P_{FPCN} and age was not significant after multiple comparison correction (β=0.27 (0.15, 0.37), *p*=0.071).

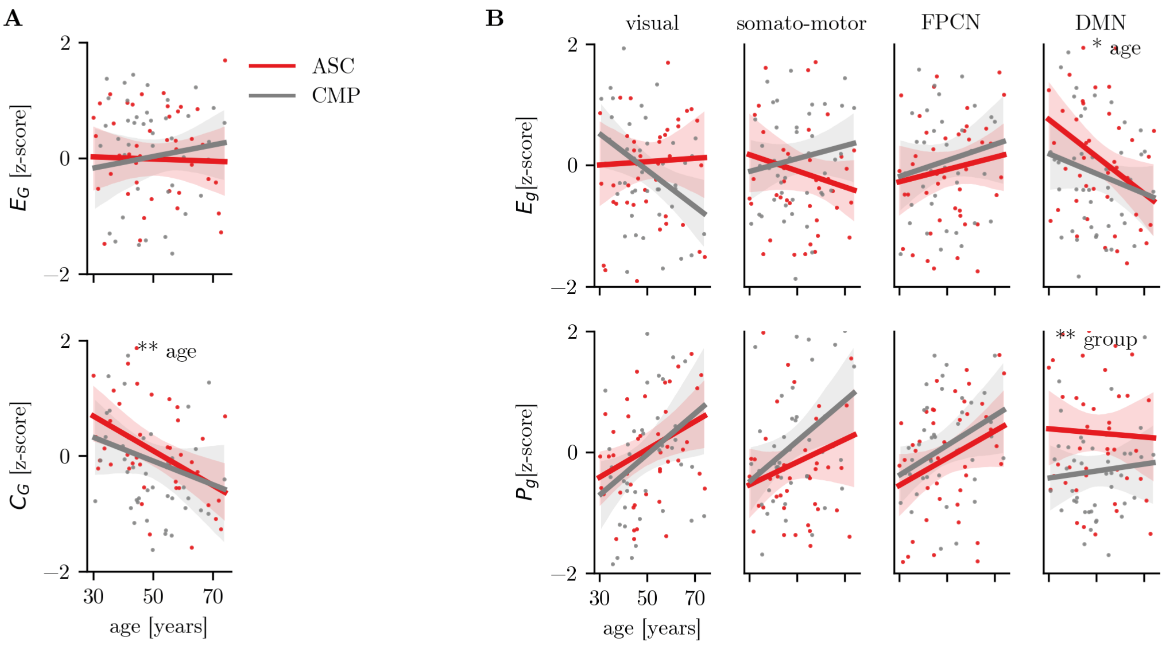

Figure S1 *Graph analysis based on the largest connected component in each network. A: Global graph metrics. B: Module-level graph metrics. Legend: * p<0.05, ** p<0.01*

Differences in graph metrics (Power et al. 2011 modules)

Using the Power et al. 2011 module solution, the results indicated a significant group x age interaction for E_g of the visual network whereby the CMP group showed lower E_g with age while there was no age-related difference in the ASD group (see **Error! Reference source not found.**C, age x group: β=-0.45 (-0.66, -0.28), *p*=0.038). Further, a decrease in E_g of the DMN was found consistent with the results obtained using the data-driven modularity solution (age: β=-0.36 (-0.49, -0.24), *p*=0.017). Analysis of P_g showed a significant increase with age for visual, somato-motor, and FPCN (visual - age: β=0.35 (0.24, 0.45), *p*=0.018; somato-motor - age: β=0.34 (0.22, 0.44), *p*=0.031; FPCN - age: β=0.32 (0.22, 0.45), *p*=0.024). Further, a group difference in P_g of the DMN was indicated similar to the results obtained with the data-driven modularity solution (group: β=-0.54 (-0.71, -0.37), *p*=0.009). There were no other significant effects of age, participant group, or the age x group interaction for E_g or P_g for any other module. Similar to the results obtained with the data-driven module assignment, there was a significant difference in the association between sdRT and P_g of the visual network (ASD: r=-0.23, CMP: r=0.37, Δz=-2.71, *p*=0.007).

References

1. Mooney C, Ferguson G (1951): A new closure test. *Can J Psychology Revue Can De Psychologie*. 5: 129.

2. Grützner C, Uhlhaas PJ, Genc E, Kohler A, Singer W, Wibral M (2010): Neuroelectromagnetic Correlates of Perceptual Closure Processes. *J Neurosci*. 30: 8342–8352.

3. Tustison NJ, Avants BB, Cook PA, Gee JC (2010): N4ITK: Improved N3 bias correction with robust B-spline approximation. *2010 Ieee Int Symposium Biomed Imaging Nano Macro*. 708–711.

4. Dale AM, Fischl B, Sereno MI (1999): Cortical Surface-Based Analysis I. Segmentation and Surface Reconstruction. *Neuroimage*. 9: 179–194.

5. Klein A, Ghosh SS, Bao FS, Giard J, Häme Y, Stavsky E, *et al.* (2017): Mindboggling morphometry of human brains. *Plos Comput Biol*. 13: e1005350.

6. Fonov V, Evans AC, Botteron K, Almli RC, McKinstry RC, Collins LD, Group B (2011): Unbiased average age-appropriate atlases for pediatric studies. *Neuroimage*. 54: 313 327.

7. Avants B, Epstein C, Grossman M, Gee J (2008): Symmetric diffeomorphic image registration with cross-correlation: evaluating automated labeling of elderly and neurodegenerative brain. *Med Image Anal*. 12: 26 41.

8. Zhang Y, Brady M, Smith S (2001): Segmentation of Brain MR Images Through a Hidden Markov Random Field Model and the Expectation-Maximization Algorithm. *Ieee T Med Imaging*. 20: 45.

9. Greve DN, Fischl B (2009): Accurate and robust brain image alignment using boundary-based registration. *Neuroimage*. 48.

10. Jenkinson M, Bannister P, Brady M, Smith S (2002): Improved optimization for the robust and accurate linear registration and motion correction of brain images. *NeuroImage*. 17: 825 841.

11. Power JD, Mitra A, Laumann TO, Snyder AZ, Schlaggar BL, Petersen SE (2014): Methods to detect, characterize, and remove motion artifact in resting state fMRI. *Neuroimage*. 84: 320–341.

12. Geerligs L, Rubinov M, Cam-CAN, Henson RN (2015): State and Trait Components of Functional Connectivity: Individual Differences Vary with Mental State. *J Neurosci*. 35: 13949–13961.

13. Geerligs L, Renken RJ, Saliasi E, Maurits NM, Lorist MM (2015): A Brain-Wide Study of Age-Related Changes in Functional Connectivity. *Cereb Cortex*. 25: 1987–1999.

14. Blondel VD, Guillaume J-L, Lambiotte R, Lefebvre E (2008): Fast unfolding of communities in large networks. *J Statistical Mech Theory Exp*. 2008: P10008.

15. Kernighan B, Lin S (2013): An Efficient Heuristic Procedure for Partitioning Graphs. *Bell Syst Technical J*. 49: 291 307.
